# 4-[(2-Chloro-4-nitro­phen­yl)diazen­yl]-*N*,*N*-diethyl­aniline

**DOI:** 10.1107/S160053681100537X

**Published:** 2011-02-19

**Authors:** Liangyu Gong, Lihua Lu

**Affiliations:** a College of Chemical & Pharmaceutical Sciences, Qingdao Agriculture University, Qingdao 266109, People’s Republic of China

## Abstract

In the title compound, C_16_H_17_ClN_4_O_2_, the aromatic ring is twisted slightly with respect to the plane of the diazene group [N—N—C—C torsion angle = −3.9 (4)°]. The NO_2_ group is twisted by 16.2 (4)° relative to the aromatic ring. The two ethyl chains are positioned such that one ethyl chain lies above and the other below the ring.

## Related literature

For background to disperse dyes, see: Freeman & Posey (1992 ▶); Freeman *et al.* (1997 ▶). For related structures, see: He *et al.* (2009 ▶); Maginn *et al.* (1993 ▶).
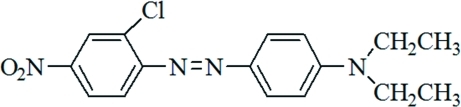


## Experimental

### 

#### Crystal data

C_16_H_17_ClN_4_O_2_
*M* *_r_* = 332.79
Monoclinic, 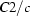

*a* = 25.745 (2) Å
*b* = 7.5774 (6) Å
*c* = 18.8628 (15) Å
β = 121.795 (5)°
*V* = 3127.6 (4) Å^3^
*Z* = 8
Mo *K*α radiation
μ = 0.26 mm^−1^
*T* = 110 K
0.40 × 0.06 × 0.04 mm

#### Data collection

Bruker–Nonius X8 APEXII diffractometer
Absorption correction: multi-scan (*SADABS*; Sheldrick, 1996 ▶) *T*_min_ = 0.903, *T*_max_ = 0.990
21470 measured reflections
3105 independent reflections
1987 reflections with *I* > 2σ(*I*)
*R*_int_ = 0.062

#### Refinement

*R*[*F* ^2^ > 2σ(*F* ^2^)] = 0.044
*wR*(*F* ^2^) = 0.132
*S* = 1.03
3105 reflections
276 parameters
All H-atom parameters refined
Δρ_max_ = 0.30 e Å^−3^
Δρ_min_ = −0.37 e Å^−3^

### 

Data collection: *APEX2* (Bruker, 2006 ▶); cell refinement: *SAINT* (Bruker, 2006 ▶); data reduction: *SAINT*; program(s) used to solve structure: *SIR92* (Altomare *et al.*, 1994 ▶); program(s) used to refine structure: *SHELXL97* (Sheldrick, 2008 ▶); molecular graphics: *XP* in *SHELXTL* (Sheldrick, 2008 ▶); software used to prepare material for publication: *SHELXTL*.

## Supplementary Material

Crystal structure: contains datablocks I, global. DOI: 10.1107/S160053681100537X/ng5115sup1.cif

Structure factors: contains datablocks I. DOI: 10.1107/S160053681100537X/ng5115Isup2.hkl

Additional supplementary materials:  crystallographic information; 3D view; checkCIF report

## Figures and Tables

**Fig. 1.**
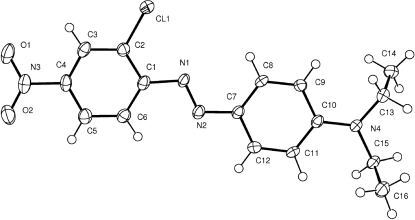
ORTEP drawing of 4-(N,N-diethylamino)-2'-chloro-4'-nitroazobenzene showing 50% probability ellipsoids.

## supplementary crystallographic information

### Comment

It is important to investigate the structural properties of disperse dyes in solid state because the absorption and dyeing performance such as dye uptake by the fibers are dependent not only on the conformation of the solid dye but also on the interactions between fiber surface and dye in molecular level. Here, we report the crystal structure of 4-(*N*,*N*-diethylamino)-2'-chloro-4'-nitroazobenzene.

In the title compound, C_16_H_17_ClN_4_O_2_, the aromatic rings in the azobenzene skeleton is essentially planar with respect to the plane of the azo group, although the N1—N2—C7—C8 torsion angle is -3.9 (4) °. The NO_2_ group is twisted relative to the aryl group to which it is bonded by 16.2 (4)° (O1—N3—C4—C3). In the *N*,*N*-diethylamino group, two ethyl chains tend to be sepatated as far apart as possible with one ethyl being above the aminobenzene plane and another one below both it.

### Experimental

The crystal was obtained by dissolving 0.5 g title compound in 50 ml acetone at room temperature and the resulting solution was covered with Parafilm plastic containing pin holes for slow evaporation of the solvent.

### Refinement

The hydrogen atoms were refined freely.

### Crystal data

**Table:**

C_16_H_17_ClN_4_O_2_	*F*(000) = 1392
*M**_r_* = 332.79	*D*_x_ = 1.413 Mg m^−^^3^
Monoclinic, *C*2/*c*	Mo *K*α radiation, λ = 0.71070 Å
*a* = 25.745 (2) Å	Cell parameters from 4405 reflections
*b* = 7.5774 (6) Å	θ = 2.5–25.5°
*c* = 18.8628 (15) Å	µ = 0.26 mm^−^^1^
β = 121.795 (5)°	*T* = 110 K
*V* = 3127.6 (4) Å^3^	Prism, red
*Z* = 8	0.40 × 0.06 × 0.04 mm

### Data collection

**Table:**

Bruker–Nonius X8 APEXII diffractometer	3105 independent reflections
Radiation source: fine-focus sealed tube	1987 reflections with *I* > 2σ(*I*)
graphite	*R*_int_ = 0.062
φ and ω scans	θ_max_ = 26.1°, θ_min_ = 1.9°
Absorption correction: multi-scan (*SADABS*; Sheldrick, 1996)	*h* = −31→31
*T*_min_ = 0.903, *T*_max_ = 0.990	*k* = −9→8
21470 measured reflections	*l* = −23→23

### Refinement

**Table:**

Refinement on *F*^2^	Primary atom site location: structure-invariant direct methods
Least-squares matrix: full	Secondary atom site location: difference Fourier map
*R*[*F*^2^ > 2σ(*F*^2^)] = 0.044	Hydrogen site location: inferred from neighbouring sites
*wR*(*F*^2^) = 0.132	All H-atom parameters refined
*S* = 1.03	*w* = 1/[σ^2^(*F*_o_^2^) + (0.0616*P*)^2^ + 3.7461*P*] where *P* = (*F*_o_^2^ + 2*F*_c_^2^)/3
3105 reflections	(Δ/σ)_max_ < 0.001
276 parameters	Δρ_max_ = 0.30 e Å^−^^3^
0 restraints	Δρ_min_ = −0.37 e Å^−^^3^

### Special details

**Table:**

Geometry. All e.s.d.'s (except the e.s.d. in the dihedral angle between two l.s. planes) are estimated using the full covariance matrix. The cell e.s.d.'s are taken into account individually in the estimation of e.s.d.'s in distances, angles and torsion angles; correlations between e.s.d.'s in cell parameters are only used when they are defined by crystal symmetry. An approximate (isotropic) treatment of cell e.s.d.'s is used for estimating e.s.d.'s involving l.s. planes.
Refinement. Refinement of *F*^2^ against ALL reflections. The weighted *R*-factor *wR* and goodness of fit *S* are based on *F*^2^, conventional *R*-factors *R* are based on *F*, with *F* set to zero for negative *F*^2^. The threshold expression of *F*^2^ > σ(*F*^2^) is used only for calculating *R*-factors(gt) *etc*. and is not relevant to the choice of reflections for refinement. *R*-factors based on *F*^2^ are statistically about twice as large as those based on *F*, and *R*- factors based on ALL data will be even larger.

### Fractional atomic coordinates and isotropic or equivalent isotropic displacement parameters (Å^2^)

**Table:**

	*x*	*y*	*z*	*U*_iso_*/*U*_eq_	
Cl1	0.05101 (3)	0.47275 (10)	0.39144 (4)	0.0324 (2)	
O1	−0.16973 (10)	0.5393 (3)	0.13154 (12)	0.0404 (5)	
O2	−0.23165 (9)	0.4835 (3)	0.17351 (13)	0.0406 (6)	
N1	0.01886 (10)	0.2751 (3)	0.49659 (12)	0.0221 (5)	
N2	0.00367 (10)	0.1881 (3)	0.54089 (13)	0.0235 (5)	
N3	−0.18043 (11)	0.4861 (3)	0.18396 (14)	0.0312 (6)	
C1	−0.03294 (12)	0.3220 (3)	0.41830 (15)	0.0230 (6)	
C2	−0.02263 (12)	0.4170 (3)	0.36330 (16)	0.0232 (6)	
C3	−0.07057 (13)	0.4702 (4)	0.28604 (16)	0.0254 (6)	
H3	−0.0649 (13)	0.545 (4)	0.2500 (19)	0.034 (8)*	
C4	−0.12889 (12)	0.4255 (3)	0.26414 (15)	0.0258 (6)	
C5	−0.14133 (13)	0.3303 (4)	0.31635 (16)	0.0268 (6)	
H5	−0.1831 (14)	0.302 (4)	0.3003 (18)	0.034 (8)*	
C6	−0.09304 (12)	0.2792 (4)	0.39309 (17)	0.0248 (6)	
H6	−0.0984 (13)	0.214 (4)	0.4299 (18)	0.035 (8)*	
C7	0.05212 (12)	0.1399 (3)	0.61996 (15)	0.0213 (6)	
C8	0.11401 (12)	0.1691 (3)	0.65168 (16)	0.0205 (6)	
H8	0.1255 (12)	0.223 (4)	0.6154 (16)	0.029 (7)*	
C9	0.15720 (13)	0.1204 (3)	0.73204 (16)	0.0220 (6)	
H9	0.1982 (14)	0.138 (4)	0.7512 (17)	0.035 (8)*	
C10	0.14028 (11)	0.0425 (3)	0.78528 (15)	0.0204 (6)	
C11	0.07782 (12)	0.0103 (4)	0.75163 (16)	0.0236 (6)	
H11	0.0638 (12)	−0.040 (3)	0.7845 (17)	0.026 (7)*	
C12	0.03514 (13)	0.0575 (4)	0.67093 (17)	0.0241 (6)	
H12	−0.0068 (13)	0.034 (3)	0.6497 (17)	0.025 (7)*	
N4	0.18316 (9)	−0.0024 (3)	0.86547 (13)	0.0233 (5)	
C13	0.24663 (12)	0.0550 (4)	0.90619 (17)	0.0251 (6)	
H13A	0.2599 (11)	0.074 (3)	0.9655 (17)	0.019 (7)*	
H13B	0.2482 (12)	0.170 (4)	0.8836 (16)	0.023 (7)*	
C14	0.28708 (14)	−0.0772 (4)	0.8975 (2)	0.0293 (7)	
H14A	0.2819 (14)	−0.196 (5)	0.917 (2)	0.053 (10)*	
H14B	0.3270 (15)	−0.039 (4)	0.9279 (18)	0.031 (8)*	
H14C	0.2750 (13)	−0.088 (4)	0.8404 (19)	0.030 (8)*	
C15	0.16615 (14)	−0.0922 (4)	0.91908 (17)	0.0271 (6)	
H15A	0.2026 (13)	−0.159 (4)	0.9614 (18)	0.034 (8)*	
H15B	0.1330 (11)	−0.175 (3)	0.8853 (15)	0.015 (6)*	
C16	0.14643 (15)	0.0348 (5)	0.96314 (19)	0.0341 (7)	
H16A	0.1835 (15)	0.110 (4)	1.005 (2)	0.047 (9)*	
H16B	0.1328 (14)	−0.027 (4)	0.994 (2)	0.044 (9)*	
H16C	0.1166 (13)	0.112 (4)	0.9259 (18)	0.029 (8)*	

### Atomic displacement parameters (Å^2^)

**Table:**

	*U*^11^	*U*^22^	*U*^33^	*U*^12^	*U*^13^	*U*^23^
Cl1	0.0281 (4)	0.0469 (4)	0.0259 (4)	0.0027 (3)	0.0167 (3)	0.0077 (3)
O1	0.0482 (14)	0.0484 (13)	0.0190 (11)	0.0144 (10)	0.0140 (10)	0.0060 (9)
O2	0.0262 (12)	0.0426 (13)	0.0366 (12)	0.0003 (9)	0.0053 (10)	0.0060 (10)
N1	0.0257 (12)	0.0269 (12)	0.0140 (11)	0.0030 (9)	0.0106 (10)	0.0017 (9)
N2	0.0266 (13)	0.0275 (13)	0.0165 (11)	0.0023 (9)	0.0113 (10)	−0.0005 (9)
N3	0.0362 (15)	0.0270 (13)	0.0199 (13)	0.0060 (10)	0.0074 (11)	−0.0003 (10)
C1	0.0290 (15)	0.0255 (14)	0.0153 (13)	0.0025 (11)	0.0122 (12)	−0.0029 (11)
C2	0.0236 (14)	0.0273 (15)	0.0194 (14)	0.0027 (11)	0.0117 (12)	−0.0007 (11)
C3	0.0348 (16)	0.0240 (14)	0.0195 (14)	0.0058 (12)	0.0158 (13)	0.0015 (11)
C4	0.0274 (15)	0.0278 (15)	0.0151 (13)	0.0070 (11)	0.0064 (12)	−0.0015 (11)
C5	0.0280 (16)	0.0278 (15)	0.0221 (15)	0.0012 (12)	0.0115 (13)	−0.0028 (12)
C6	0.0297 (16)	0.0265 (15)	0.0206 (14)	0.0008 (12)	0.0147 (13)	−0.0013 (12)
C7	0.0258 (15)	0.0226 (14)	0.0174 (13)	0.0031 (11)	0.0127 (12)	0.0003 (10)
C8	0.0272 (15)	0.0214 (13)	0.0184 (14)	0.0005 (10)	0.0158 (12)	0.0009 (10)
C9	0.0209 (14)	0.0262 (14)	0.0206 (14)	−0.0018 (11)	0.0121 (12)	−0.0015 (11)
C10	0.0249 (14)	0.0221 (13)	0.0168 (13)	0.0042 (11)	0.0127 (11)	0.0004 (10)
C11	0.0262 (15)	0.0323 (15)	0.0191 (14)	0.0010 (11)	0.0165 (12)	0.0020 (11)
C12	0.0225 (15)	0.0307 (15)	0.0233 (15)	−0.0004 (12)	0.0149 (13)	0.0003 (12)
N4	0.0223 (12)	0.0320 (13)	0.0171 (11)	0.0015 (9)	0.0114 (10)	0.0049 (9)
C13	0.0256 (15)	0.0308 (16)	0.0179 (14)	0.0004 (12)	0.0107 (12)	0.0020 (12)
C14	0.0247 (17)	0.0366 (18)	0.0286 (17)	0.0031 (13)	0.0156 (14)	0.0059 (13)
C15	0.0279 (16)	0.0371 (17)	0.0191 (14)	0.0053 (13)	0.0143 (13)	0.0090 (12)
C16	0.0328 (18)	0.0495 (19)	0.0223 (16)	0.0079 (16)	0.0161 (15)	0.0034 (15)

### Geometric parameters (Å, °)

**Table:**

Cl1—C2	1.731 (3)	C9—H9	0.93 (3)
O1—N3	1.226 (3)	C10—N4	1.365 (3)
O2—N3	1.226 (3)	C10—C11	1.405 (4)
N1—N2	1.276 (3)	C11—C12	1.375 (4)
N1—C1	1.418 (3)	C11—H11	0.95 (3)
N2—C7	1.398 (3)	C12—H12	0.95 (3)
N3—C4	1.464 (3)	N4—C13	1.458 (3)
C1—C6	1.397 (4)	N4—C15	1.465 (3)
C1—C2	1.398 (4)	C13—C14	1.514 (4)
C2—C3	1.384 (4)	C13—H13A	0.99 (3)
C3—C4	1.373 (4)	C13—H13B	0.98 (3)
C3—H3	0.95 (3)	C14—H14A	1.01 (3)
C4—C5	1.388 (4)	C14—H14B	0.92 (3)
C5—C6	1.376 (4)	C14—H14C	0.96 (3)
C5—H5	0.98 (3)	C15—C16	1.523 (4)
C6—H6	0.92 (3)	C15—H15A	0.99 (3)
C7—C8	1.393 (4)	C15—H15B	0.98 (3)
C7—C12	1.396 (4)	C16—H16A	1.03 (3)
C8—C9	1.376 (4)	C16—H16B	0.95 (3)
C8—H8	0.97 (3)	C16—H16C	0.93 (3)
C9—C10	1.417 (4)		
			
N2—N1—C1	111.5 (2)	C11—C10—C9	117.3 (2)
N1—N2—C7	115.1 (2)	C12—C11—C10	120.8 (2)
O1—N3—O2	123.7 (2)	C12—C11—H11	118.2 (17)
O1—N3—C4	118.0 (2)	C10—C11—H11	120.9 (17)
O2—N3—C4	118.3 (2)	C11—C12—C7	121.4 (3)
C6—C1—C2	118.4 (2)	C11—C12—H12	119.1 (16)
C6—C1—N1	124.2 (2)	C7—C12—H12	119.5 (16)
C2—C1—N1	117.4 (2)	C10—N4—C13	122.5 (2)
C3—C2—C1	121.3 (3)	C10—N4—C15	121.3 (2)
C3—C2—Cl1	118.5 (2)	C13—N4—C15	115.8 (2)
C1—C2—Cl1	120.2 (2)	N4—C13—C14	112.9 (2)
C4—C3—C2	118.2 (3)	N4—C13—H13A	105.2 (15)
C4—C3—H3	119.1 (18)	C14—C13—H13A	111.6 (14)
C2—C3—H3	122.5 (18)	N4—C13—H13B	109.2 (15)
C3—C4—C5	122.6 (2)	C14—C13—H13B	110.5 (15)
C3—C4—N3	119.0 (2)	H13A—C13—H13B	107 (2)
C5—C4—N3	118.3 (2)	C13—C14—H14A	108.4 (18)
C6—C5—C4	118.3 (3)	C13—C14—H14B	109.2 (18)
C6—C5—H5	120.2 (17)	H14A—C14—H14B	112 (3)
C4—C5—H5	121.5 (17)	C13—C14—H14C	110.1 (17)
C5—C6—C1	121.2 (3)	H14A—C14—H14C	109 (2)
C5—C6—H6	122.2 (18)	H14B—C14—H14C	109 (2)
C1—C6—H6	116.6 (18)	N4—C15—C16	113.0 (3)
C8—C7—C12	118.5 (2)	N4—C15—H15A	107.0 (16)
C8—C7—N2	126.3 (2)	C16—C15—H15A	109.2 (16)
C12—C7—N2	115.1 (2)	N4—C15—H15B	108.9 (14)
C9—C8—C7	120.5 (2)	C16—C15—H15B	109.6 (14)
C9—C8—H8	121.5 (16)	H15A—C15—H15B	109 (2)
C7—C8—H8	118.0 (16)	C15—C16—H16A	108.9 (18)
C8—C9—C10	121.4 (2)	C15—C16—H16B	111.3 (19)
C8—C9—H9	118.9 (18)	H16A—C16—H16B	107 (3)
C10—C9—H9	119.7 (18)	C15—C16—H16C	111.5 (17)
N4—C10—C11	121.5 (2)	H16A—C16—H16C	107 (3)
N4—C10—C9	121.2 (2)	H16B—C16—H16C	110 (3)
			
C1—N1—N2—C7	−178.7 (2)	N1—N2—C7—C8	−3.9 (4)
N2—N1—C1—C6	0.3 (4)	N1—N2—C7—C12	175.0 (2)
N2—N1—C1—C2	−179.6 (2)	C12—C7—C8—C9	−1.2 (4)
C6—C1—C2—C3	0.9 (4)	N2—C7—C8—C9	177.7 (2)
N1—C1—C2—C3	−179.1 (2)	C7—C8—C9—C10	−1.1 (4)
C6—C1—C2—Cl1	−179.81 (19)	C8—C9—C10—N4	−178.7 (2)
N1—C1—C2—Cl1	0.1 (3)	C8—C9—C10—C11	2.6 (4)
C1—C2—C3—C4	−0.7 (4)	N4—C10—C11—C12	179.5 (2)
Cl1—C2—C3—C4	180.0 (2)	C9—C10—C11—C12	−1.8 (4)
C2—C3—C4—C5	0.2 (4)	C10—C11—C12—C7	−0.5 (4)
C2—C3—C4—N3	177.8 (2)	C8—C7—C12—C11	2.0 (4)
O1—N3—C4—C3	16.2 (4)	N2—C7—C12—C11	−177.0 (2)
O2—N3—C4—C3	−162.6 (2)	C11—C10—N4—C13	−169.7 (2)
O1—N3—C4—C5	−166.1 (2)	C9—C10—N4—C13	11.6 (4)
O2—N3—C4—C5	15.0 (4)	C11—C10—N4—C15	2.3 (4)
C3—C4—C5—C6	0.0 (4)	C9—C10—N4—C15	−176.4 (2)
N3—C4—C5—C6	−177.5 (2)	C10—N4—C13—C14	−92.7 (3)
C4—C5—C6—C1	0.2 (4)	C15—N4—C13—C14	94.9 (3)
C2—C1—C6—C5	−0.7 (4)	C10—N4—C15—C16	−85.4 (3)
N1—C1—C6—C5	179.4 (2)	C13—N4—C15—C16	87.1 (3)
